# Enhancement of Power Output by using Alginate Immobilized Algae in Biophotovoltaic Devices

**DOI:** 10.1038/s41598-017-16530-y

**Published:** 2017-11-24

**Authors:** Fong-Lee Ng, Siew-Moi Phang, Vengadesh Periasamy, Kamran Yunus, Adrian C. Fisher

**Affiliations:** 1 Institute of Ocean and Earth Sciences (IOES), University of Malaya, 50603 Kuala Lumpur, Malaysia; 2 Institute of Biological Sciences, Faculty of Science, University of Malaya, 50603 Kuala Lumpur, Malaysia; 3 Low Dimensional Materials Research Centre (LDMRC), Department of Physics, University of Malaya, 50603 Kuala Lumpur, Malaysia; 4 Department of Chemical Engineering and Biotechnology, University of Cambridge, Philipa Fawcett Drive, CB3 0AS Cambridge, United Kingdom

## Abstract

We report for the first time a photosynthetically active algae immobilized in alginate gel within a fuel cell design for generation of bioelectricity. The algal-alginate biofilm was utilized within a biophotovoltaics (BPV) device developed for direct bioelectricity generation from photosynthesis. A peak power output of 0.289 mWm^−2^ with an increase of 18% in power output compared to conventional suspension culture BPV device was observed. The increase in maximum power density was correlated to the maximum relative electron transport rate (rETRm). The semi-dry type of photosynthetically active biofilm proposed in this work may offer significantly improved performances in terms of fuel cell design, bioelectricity generation, oxygen production and CO_2_ reduction.

## Introduction

Light has been used as an energy input in a variety of photovoltaic devices. Wu *et al*. (2011) produced a near-infrared laser-driven organic photovoltaic device (OPV), which can convert laser light to electrical power using a blend of poly(3-hexylthiophene) (P3HT) and [6,6]-phenyl-C61-butyric acid methyl ester (PCBM) with maximum power output of 2.10 µW^1^. The performance of the OPV was enhanced based on a blend of P3HT and PCBM, through the introduction of NaYF4:Yb/Er NCs with maximum power output of 9.05 µW^2^. Dye-sensitized photovoltaic devices generating a maximum power output of 22.2 µW when illuminated by 980 nm laser light, have been suggested as a potential electrical source for powering nanodevices under the skin^3^. Recently, Hsiao *et al*. (2016) developed a biocompatible OPV using low-toxicity β-carotene and perylene materials which, when stimulated with a white light LED, generated 35.34 µW power output^4^. Microorganisms such as cyanobacteria and microalgae that carry out conventional and bacterial photosynthesis, have high resilience and can live in a wide range of conditions from high temperatures to low light conditions^5^. The cultivation of these photosynthetic organisms for producing sustainable fuels and chemical feedstock is on the increase^6^. Microalgae are amongst the most efficient photosynthetic organisms, with fast growth rates, diverse products and tolerance to extreme environments^5^. These photosynthetic organisms successfully harvest solar energy and convert this energy into chemical energy^7,8^, and store this energy in the form of oils, carbohydrates and proteins^9^. Recent studies have reported the use of microalgae in fuel cells (FCs), giving rise to a novel range of systems based on biological photovoltaic devices or BPVs^10,11^. Photo-microbial fuel cells have been developed based on the utilization of cyanobacteria for hydrogen generation^12^ and electricity generation using 2-hydroxy-1,4-naphthoquinone as an electron shuttle between the algae cells and a carbon-cloth anode^13^. The generation of bioelectricity directly from algal photosynthesis using biophotovoltaic (BPV) devices have been reported. Bombelli *et al*. (2011) used whole cells as well as thylakoid membranes isolated from the Cyanobacterium *Synechocystis* and generated total power output of 4.71 and 9.28 nW µmol/Chl^10^. Inglesby *et al*. (2013) generated 1.12 × 10^–4^ W m^−2^ using BPV with biofilm of another Cyanobacterium *Arthrospira* on ITO-Polyethyleneterephthalate anode^14^. Ng *et al*. (2014) used biofilms of two species of the Chorophyte *Chlorella* and two species of Cyanobacteria *Spirulina* and *Synechococcus* on ITO anodes and obtained increased power output ranging from 1.12 × 10^–4^ to 3.13 × 10^–4^ W m^−2^ ^8^. Replacing ITO with reduced graphene oxide (rGO) anode, using the Langmuir-Blodgett method, power output was further increased by 119% compared to the former type of anode^15^.

**Figure 1:**
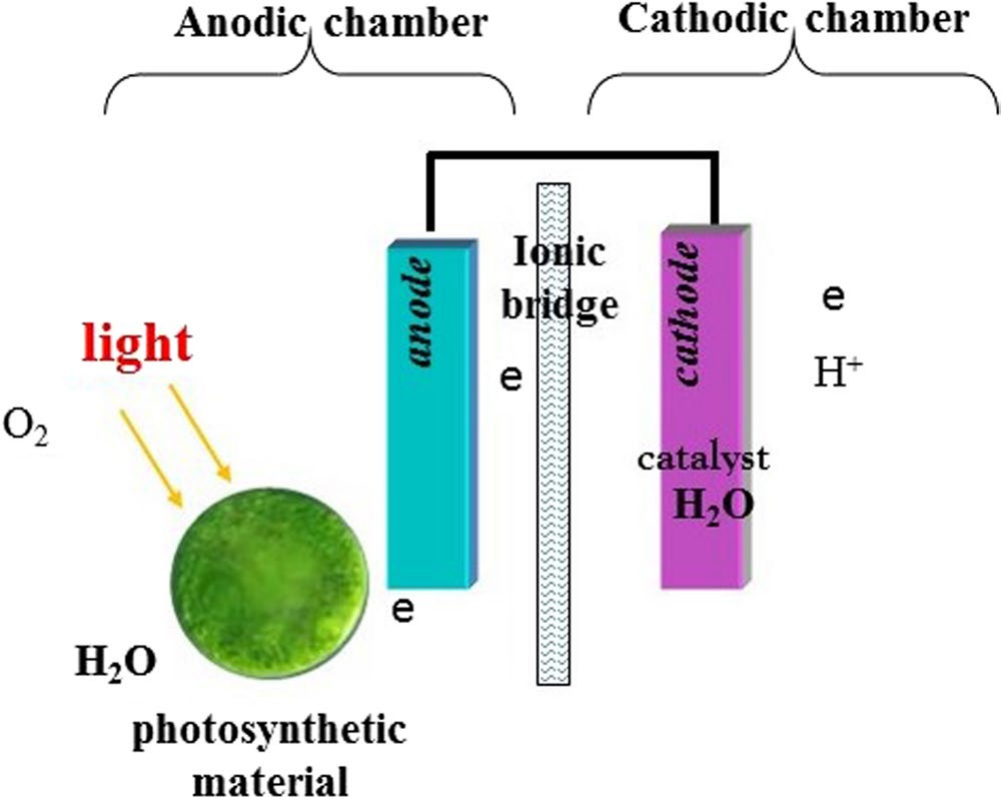
Working principle of the biophotovoltaic system.

Various biological components have been introduced into fuel cells (FCs), giving rise to biophotovoltaic devices (BPVs), BPVs produce electricity from light energy via the light harvesting apparatus of the photosynthetic organisms. In BPVs, the photosynthetic component splits water into molecular oxygen, protons and electrons using the energy of light^16,17^ (Fig. 1). A BPV with a photosynthetic component has several possible steps between the water protolysis process and the electron donation process to an anode. The electrons travel to the anode via direct cell contact through redox proteins^18^, nano wires^19^ and/or endogenous compounds such as menaquinone^20,21^ or exogenous electron transfer mediators (ETMs)^10^.

Immobilization technology in microalgal cultivation is an attractive technique to restrict the freedom of movement of algal cells in an aqueous medium by entrapping them in a solid matrix^22^. Gel entrapment is a common way to immobilize microalgal cells. Natural polysaccharides such as agar, carrageenan and alginate are suitable materials for immobilization of microalgae, because of their low toxicity and high transparency^23^.

Alginate is a natural polysaccharide obtained from the cell wall of marine brown macroalgae and is usually produced commercially as sodium or calcium alginate^24^. Alginate can also be produced by certain bacterial strains such as *Azotobacter vinelandii* and *Pseudomonas aeroginosa*. Alginate is the only polysaccharide, which naturally contains carboxyl groups in each constituent residue, and possesses abilities of gel formation in the presence of polyvalent cations, such as Ca^2+^ to produce strong gels or insoluble polymers^25^. Alginate is the most frequent polymer used for algal immobilization studies. Its advantages include ease of preparation, non-toxic, transparent, and biocompatibility^26,27^.

Yagishita *et al*. (1998) used immobilized *Anabaena variabilis* within alginate beads in photosynthetic electrochemical cells, and reported a total energy conversion of 0.2% from light energy^28^. Other studies reported that immobilized microalgae within alginate beads in microbial fuel cells can enhance power generation. The Coulombic efficiency of the microbial fuel cells with immobilized *Chlorella vulgaris* in alginate beads could reach 9.40% and 14.1%, respectively^29,30^. In the present study, we evaluated the feasibility of using alginate immobilized *Chlorella* cells coated directly on the ITO anode surface, to enhance bioelectricity generation. A comparison of power output generation was carried out between the BPV using the immobilized and algal suspension cultures.

## Results and Discussion

### Chlorophyll-a (Chl-a) content and photosynthetic performance of *Chlorella* sp

Chlorophyll a (Chl-a) concentration in cultures is a proxy for biomass and is used to indicate the growth rate of the algal cells. Pulse amplitude modulated (PAM) fluorescence measurements are now widely used as a simple, fast and non-destructive method to assess physiological status in photosynthetic organisms. Radiant energy absorbed by chlorophyll (Chl) can undergo one of three fates: (i) use in photosynthesis (chemical work) (ii) dissipation as heat or (iii) re-emission as Chl fluorescence^31^. Hence, by measuring the yield of Chl fluorescence, information about the efficiency of photochemistry and heat dissipation can be generated using a PAM fluorometer (Diving PAM, Walz, Germany)^32^. Maximum quantum efficiency of Photosystem II (Fv/Fm) is a parameter much used to investigate the physiological state of phytoplankton and was employed here to indicate if the cells were stressed. Rapid light curves generated from PAM provide details on the relationship between photosynthetic electron transport and light intensity and generated the following parameters: (i) Alpha (α): is a measure of the light harvesting efficiency of the algae and is higher in more shade adapted cells. (ii) Maximum relative electron transport (rETRmax or Pmax): indicates the maximum capacity for photosynthesis. Algae grown in bright environments and/or with high rates of photosynthesis typically have high values. (iii) Ek: The photoadaptive index, indicates how well cells are adapted to their light environment. Healthy cells should have an Ek similar to their ambient light environment^33,34^.

Table 1 gives details of the Chl-a and photosynthetic performance in the BPV devices with immobilized and suspension cultures of *Chlorella* sp. Chl-a increased with time, indicating the growth of the algae, which reached maximum on day 12. The specific growth based on Chl-a for the immobilized and suspension cultures were 0.66 and 0.48 day^−1^, respectively. The immobilized algae grew faster because of the higher irradiance available to the thin layer of gel biofilm. On day 0, the *Chlorella* cells in the immobilized state exhibited stress, due to a change in the physical environment, as shown by the lower F_v_/F_m_ values (0.18 ± 0.01) compared to the suspension cell culture (0.25 ± 0.01) (Table 1). As the cultures grew, the physiological health of the immobilized cells improved up to day 8; the Fv/Fm of the immobilized cells was 0.46 ± 0.04. However there may have been some nutrient limitation and CO_2_ diffusion limitation occurring in the immobilized cells due to high cell density and high physiological activities inside the alginate films, as the suspension cultures had a higher F_v_/F_m_ of 0.65 than immobilised cultures, suggesting that the latter may be showing signs of stress (reduction in quantum efficiency of PSII).

**Table 1:** Comparison of Chl-a and photosynthetic performance in BPV device with suspended and immobilized cultures of *Chlorella* UMACC 313; data as means ± S.D. (n = 3).

**Day**	**Chl-a (mg/L)**		**Fv/Fm**		**Alpha**		**rETRmax (µ mol electrons m** ^**−2**^ **s** ^**−1**^ **)**		**Ek (µ mol photons** ^**−2**^ **s** ^**−1**^ **)**	
	**Suspended**	**Immobilized**	**Suspended**	**Immobilized**	**Suspended**	**Immobilized**	**Suspended**	**Immobilized**	**Suspended**	**Immobilized**
**0**	0.16 ± 0.02^e^	0.14 ± 0.02^e^	0.25 ± 0.01 ^f^	0.18 ± 0.01 ^g^	0.16 ± 0.01^c^	0.09 ± 0.01^d^	49.97 ± 3.71^d^	23.14 ± 3.50^e^	322.19 ± 37.80 ^cd^	258.34 ± 42.76^d^
**4**	1.12 ± 0.08^d^	1.89 ± 0.25^c^	0.48 ± 0.04 ^cd^	0.36 ± 0.02^e^	0.26 ± 0.01^a^	0.21 ± 0.01^b^	131.26 ± 1.25^a^	68.94 ± 7.71^c^	501.15 ± 13.02^ab^	328.94 ± 38.70 ^cd^
**8**	2.28 ± 0.13^c^	2.19 ± 0.14^c^	0.65 ± 0.01^a^	0.46 ± 0.04^d^	0.25 ± 0.02^a^	0.27 ± 0.01^a^	129.86 ± 6.59^a^	131.66 ± 7.63^a^	527.55 ± 75.76^a^	498.30 ± 43.10^ab^
**12**	5.99 ± 0.31^a^	5.16 ± 0.05^b^	0.58 ± 0.01^b^	0.52 ± 0.02^c^	0.26 ± 0.02^a^	0.23 ± 0.02^ab^	68.64 ± 2.13^c^	89.89 ± 7.13^b^	268.32 ± 23.48^d^	392.73 ± 21.48^bc^

Differences between alphabets indicate significant difference between different strains (ANOVA, Tukey HSD test, p < 0.05).

Alpha was low in immobilized cells on day 0 (0.16 ± 0.01); cells in the immobilized condition took longer time to acclimatize and cope with the physical change of the environmental conditions compared to suspended cells. On day 12, the Fv/Fm (0.52 ± 0.02) of immobilized cells indicated no stress but the alpha was low (0.23 ± 0.02) suggesting that nutrient depletion may have contributed to the low alpha value. Cell density in the immobilized gel was high by day 12, so nutrient diffusion into the alginate gel might not be sufficiently rapid to support high physiological activities of the algal cells.

The relative maximum electron transport rate (rETRm) from all cultures ranged from 23.14 to 131.66 µmol electrons m^−2^s^−1^ (Table 1). The rETRm of the suspension cultures increased from day 0 till day 8 but started to decrease after that. The immobilized culture had increased rETRm with time and was higher than the suspension culture on day 8 and day 12. This shows that the immobilized algae maintained their photosynthetic efficiency till the late growth phase of day 12, while the suspension cultures experienced a reduction in photosynthetic efficiency due to stresses arising from nutrient depletion, light limitation etc.^35^. This is indicated by the higher algal biomass as indicated by the Chl-a content, in the suspension culture than the immobilized system. The photoadaptive index, E_k_ had relatively high values and ranged from 258.34 to 527.55 µmol photons^−2^s^−1^ indicating that the *Chlorella* UMACC 313 was able to adapt to high light intensities. The suspension cultures had higher E_k_ values than the immobilized culture, resulting in higher F_v_/F_m_ in the former.

### Bioelectricity generation from BPV devices with immobilized and suspension culture of *Chlorella* sp

**Figure 2:**
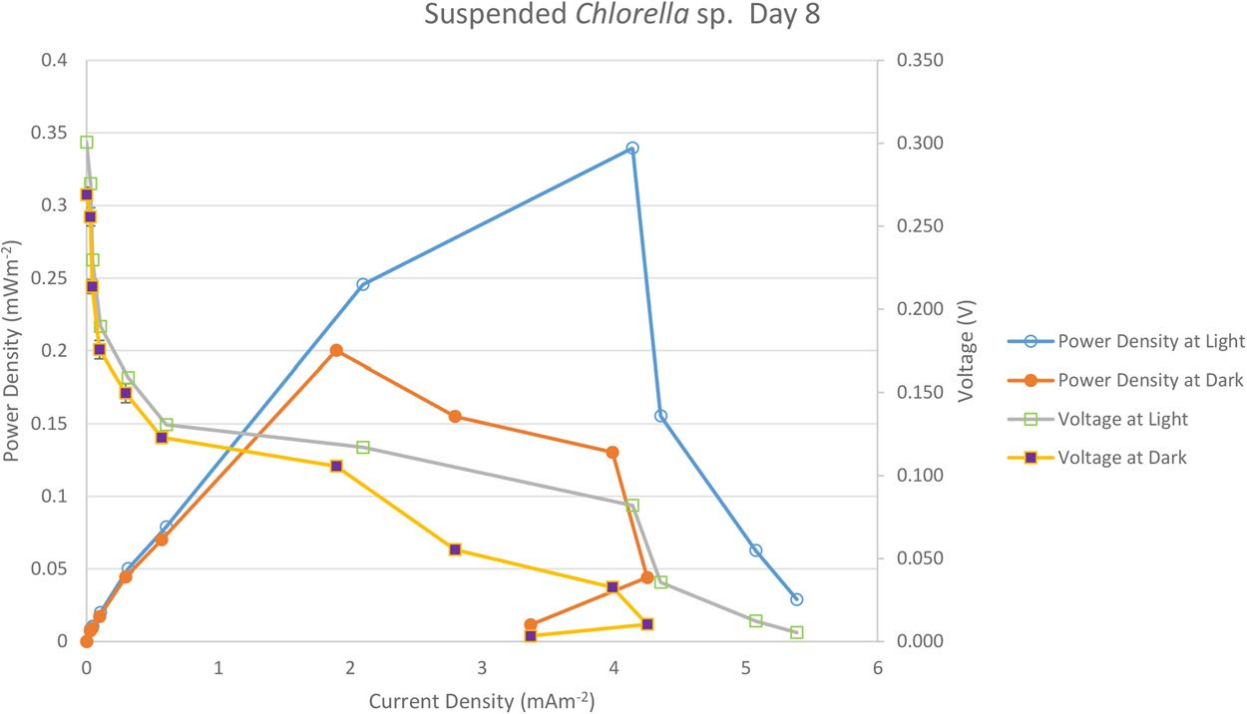
The polarization curve from suspended *Chlorella* UMACC 313 BPV platforms on day 8.

**Figure 3:**
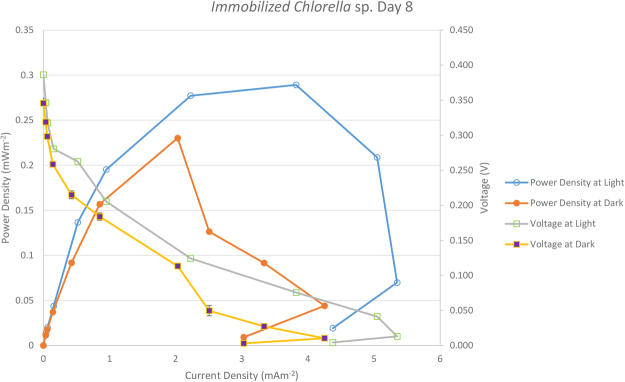
The polarization curve from immobilized *Chlorella* UMACC 313 biophotovoltaic platforms on day 8.

Table 2 gives details of the maximum current density and maximum power density in BPV devices with immobilized and suspension cell cultures of *Chlorella* UMACC 313. Figures 2 and 3 show the polarization curve from suspension and immobilized *Chlorella* BPV devices on day 8, respectively. Highest maximum power density was registered on day 8, generated from immobilized *Chlorella* under light (0.289 mWm^−2^) and dark conditions (0.230 mWm^−2^). There was an increase of 18 and 15% in power output compared to the suspension culture, under light and dark conditions, respectively. Power output based on algal biomass (using Chl-a content) was estimated as 40.80 ± 2.35 mWm^−2^/mg Chl-a generated in light condition and 32.50 ± 1.85 mWm^−2^/mg Chl-a generated in dark condition for the immobilized algae BPV devices. For the algae suspension devices, 21.77 ± 2.67 mWm^−2^/mg Chl-a in light and 17.42 ± 1.38 mWm^−2^/mg Chl-a in dark conditions. These values were obtained by dividing the maximum power density by the total Chl-a content in the BPV devices (ANOVA, Tukey HSD test, p < 0.05). Immobilized algae BPV devices registered higher power output compared to the algae suspension devices from day 0 to day 12. *Chlorella* cells were embedded in the alginate matrix and as the cells divided, compact colonies were formed. This resulted in the algal cells being very close together compared to suspension cultures. The algae alginate film was in direct contact with the ITO anode, which reduced the liquid-phase mass transfer resistance and was favourable to the electron transportation from algal cells to ITO anode^29^. Power output increased until day 8 and showed slight a decline on day 12. This might be due to an increase in cell density within the alginate film that caused self-shading and/or a decrease in nutrient diffusion efficiency that limited cell growth, and led to lower power output.

**Table 2:** Comparison of Maximum Current Density and Maximum Power Density in BPV device with suspended and immobilized culture of *Chlorella* UMACC 313; data as means ± S.D.

**Day**	**Maximum Current Density (mAm** ^**−2**^ **)**				**Maximum Power Density (mWm** ^**−2**^ **)**			
	**Suspended**		**Immobilized**		**Suspended**		**Immobilized**	
	**Light**	**Dark**	**Light**	**Dark**	**Light**	**Dark**	**Light**	**Dark**
**0**	1.509 ± 0.238^ef^	1.097 ± 0.237 ^f^	1.684 ± 0.517^ef^	1.508 ± 0.238^ef^	0.026 ± 0.001^fg^	0.017 ± 0.001 ^g^	0.073 ± 0.007^de^	0.062 ± 0.004^def^
**4**	1.751 ± 0.145^def^	1.684 ± 0.583^ef^	2.189 ± 0.058^de^	1.700 ± 0.077^ef^	0.074 ± 0.007^de^	0.048 ± 0.005^efg^	0.095 ± 0.005^d^	0.067 ± 0.003^de^
**8**	5.387 ± 0.583^a^	3.367 ± 0.583^bc^	5.349 ± 0.412^a^	4.252 ± 0.238^b^	0.245 ± 0.019^b^	0.200 ± 0.009^c^	0.289 ± 0.004^a^	0.230 ± 0.008^bc^
**12**	2.768 ± 0.254 ^cd^	1.751 ± 0.204^def^	3.541 ± 0.211^bc^	2.523 ± 0.186^cde^	0.155 ± 0.009^c^	0.095 ± 0.007^d^	0.201 ± 0.285^c^	0.119 ± 0.001 ^g^

(n = 3). Differences between alphabets indicate significant difference between different strains (ANOVA, Tukey HSD test, p < 0.05).

Further work is currently being carried out to determine the limiting factors and optimise the system. The highest power output was registered on day 8 in the BPV devices with immobilized cell cultures. During the experiments, the BPV devices were maintained at an irradiance of 30 µmol photons m^−2^ s^−1^. From the rapid light curves (RLC), the electron transport rates (ETR) at the exposure irradiance of 30 µmol photons m^−2^s^−1^ were 64.36 µmol electrons m^−2^s^−1^ and 116.20 µmol electronsm^−2^s^−1^ for BPV devices with suspended and immobilized cell cultures respectively. Thus the BPV devices using immobilized cell cultures registered an increase of 80.53% in ETR at the exposure irradiance compared to the conventional BPV device with suspended cultures. This explained why the power output from the immobilized device is higher than that of suspended device on day 8. We previously reported continued power output under dark condition in the algal BPV using the same *Chlorella* sp. on a rGO anode^15^. This was explained by metabolism of organic substrates stored within or secreted by the cells during the dark phase. The ability to store metabolites inside the algal cell may allow power generation in the dark^36^. Sekar *et al*. (2014) used a cyanobacterium *Nostoc* sp. as a photo-biocatalyst on the anode of photo-bioelectrochemical cells and reported a lower current generation in dark condition, probably due to a shut off in the light driven electron transport reactions in the linear photosynthetic pathway^37^.

Live algal cells function as electron donors. The electron transport rate determines the power output. In the immobilized cell system, each algal cell starts to divide and form colonies within the gel matrix, as opposed to the suspension culture where the cells may be separated in the aqueous medium. The colonial property could potentially give good connectivity between the algal cells in the immobilized system, allowing for possible faster electron transport from cell to cell and to the anode. This then contributes to the higher rETR_m_ and the higher power output. This is the first time that such a phenomenon was observed and reported.

Sodium alginate is a linear chained polysaccharide and natural anionic biopolymer extracted from brown algae. It consists of two uronic acids, composed of 1,4-linked β-D-mannuronic acid (M block units) and α-l-guluronic acid (G block units) arranged in an irregular blockwise pattern forming homopolymeric MM or GG blocks and heteropolymeric MG or GM blocks^38^. Liu *et al*. (2014) reported high proton conductivity of 5.5 × 10^−3^ s/cm in sodium alginate film with relative humidity of 57% at room temperature^39^. Li *et al*. (2012) using sodium alginate gel as a binder for lithium ion batteries. A large number of carboxylic group in sodium alginate leads to a greater number of possible binder-bonds for electrode materials and excellent mechanical properties in electrolyte solvents^40^. Kovalenko *et al*. (2014) reported that a mixture of Si nanopowder and sodium alginate can become a stable anode with reversible capacity eight times higher than a graphite anode^41^. In alginate, carboxylic groups are naturally present and are evenly distributed in the polymer chain; this is different compared to the synthetic polymers such as carboxymethyl cellulose. The latter has random distribution of carboxylic groups, where some monomeric units may have more than one carboxylic groups. The higher concentration and uniform distribution of carboxylic groups in alginate contributed to better contact with the microalgae cells and ITO anode in this study. Besides, macromolecules in alginate are much more polar compared to synthetic polymers, which contribute to better interfacial interaction between the polymer binder with the microalgae cells and at the same time ensure stronger adhesion to the ITO anode^41^. This may have resulted in enhanced electron transfer from immobilized algae to the anode.

## Conclusions

The immobilization of algae in alginate to form a biofilm on the anode produced 18% higher power output than suspension cell culture on the anode. This was confirmed by the higher photosynthetic performance of the immobilized cells in terms of rETRm. The immobilized culture formed colonies that possibly increased cellular connectivity to enhance electron transport in the BPV. Immobilized cells could maintain the photosynthetic efficiency longer than suspension cell culture.

It is believed that this semi-dry gel-like biofilm structures may impart significantly superior features that enables development of non-aqueous type fuel cell designs. Coupled with its possible flexible applications onto conductive glass substrates for bioelectricity and enhanced oxygen generation, this current progress in fuel cell technology may finally allow realization of “artificial leaf” technologies in future. This totally green technology enables not only power generation, but also provides a carbon negative solution towards reducing the global carbon footprint.

## Methods

### Algal culture

A local tropical algal strain from the University of Malaya Algae Culture Collection (UMACC) *Chlorella* sp. (UMACC 313) that was isolated from an anaerobic treatment pond for palm oil mill effluent, was selected for this study. An inoculum size of 20%, standardized at an OD of 0.2 at 620 nm (OD_620 nm_) from exponential phase cultures was used. The cultures were grown in Bold’s Basal Medium (BBM)^42^ in 1 L conical flasks (total volume: 500 ml) in an incubator shaker (120 rpm) at 25 °C, with the irradiance of 30 μmol photons m^−2^s^−1^on a 12:12 light:dark cycle.

### Preparation of immobilized algal on ITO coated glass

Cells of *Chlorella* UMACC 313 were immobilized using sodium alginate powder (food grade, purchased from Natural Colloids Industries Pte. Ltd). The immobilization of algae in 2% sodium alginate was modified after the method of Kiersten and Bucke (1977)^43^. 2 g of sodium alginate powder was added into 95 ml of sterile distilled water and stirred continuously using a magnetic stirrer overnight. The algal cells in logarithmic growth phase were harvested by centrifugation at 4000 rpm for 10 minutes. The cells were suspended in BBM to form a concentrated algal suspension (OD_620nm_ = 1.0). 5 ml of concentrated *Chlorella* sp. was mixed with 95 ml sodium alginate solution to form the algal alginate suspension. ITO coated glass purchased from KINTEC, Hong Kong had a layer thickness of 100 nm with 377.0 Ω/sq and about 10^4^ S/cm of sheet resistance and conductivity^15^. The ITO anode of dimension (3.5 cm × 3.5 cm) was placed in a mould made by sterile aluminium foil. 3 ml of the algal alginate suspension was spread on to the ITO coated glass using a pipette, and allowed to settle under gravity for 15 minutes. Sterile CaCl_2_ (0.1 M) was sprayed onto the surface until it was fully covered. Samples were left for one hour to complete the gelation process to form a 2 mm thick (measured with Elcometer 3230 Wet Film Wheels) algal gel film on the ITO. The ITO anode with the algal gel film was then rinsed with sterile distilled water to remove CaCl_2_. This algae immobilized anode was prepared to provide sufficient replicates to be used for the power generation at days 0, 4, 8 and 12, with 3 replicates each time. Suspension cell cultures for comparison with the immobilized cultures, were grown in the same way as above, and placed on top of the ITO anode in the BPVs.

### Chlorophyll-a concentration and growth

On days 0, 4, 8 and 12, the algae (immobilized and suspension cultures) on the anodes were removed and analysed for the Chl-a content. The Chl-a concentration was evaluated using spectrophotometric method^44^. The suspended algal culture was removed from ITO, separated and collected by a glass-fibre filter paper (Whatman GF/C, 0.45 µm) and the papers were mashed using a tissue grinder (Kimble, USA) with 10 mL of analytical grade 100% acetone. The samples were then left in a freezer (4 °C) for 24 hours before being centrifuged (3,000 rpm, 10 minutes, 4 °C). Absorption of the supernatant was measured at the wavelengths of 630 (OD_630nm_), 645 (OD_645nm_) and 665 nm (OD_665nm_). For the immobilized algae, the gel layer was removed from the anode and placed into a tissue grinder to be mashed to release the algae with 10 mL of analytical grade 100% acetone. The rest of the protocol as for the suspension culture was followed.

The Chl-a concentration was computed using the following formula:

$$\begin{array}{c}{\rm{Chl}}-{\rm{a}}({{\rm{mgm}}}^{-3})=({\rm{Ca}}\times {\rm{Va}})/{\rm{Vc}}\\ \mathrm{where},\\ {\rm{Ca}}=11.6\times {{\rm{OD}}}_{665{\rm{nm}}}-1.31\times {{\rm{OD}}}_{645{\rm{nm}}}-0.14\times {{\rm{OD}}}_{630{\rm{nm}}}\\ {\rm{Va}}={\rm{Volume}}\,{\rm{of}}\,{\rm{acetone}}\,({\rm{mL}})\,{\rm{used}}\,{\rm{for}}\,{\rm{extraction}}\\ {\rm{Vc}}={\rm{Volume}}\,{\rm{of}}\,{\rm{culture}}({\rm{L}})\\ {\rm{Chl}}-{\rm{a}}({\rm{mg}}/{\rm{L}})={\rm{Chl}}-{\rm{a}}({{\rm{mgm}}}^{-3})/1000\end{array}$$

The specific growth rate (µ) was calculated based on Chl-a content (mg/L) using the following formula^45^:

$$\begin{array}{c}{\rm{SGR}},\,\mu \,({{\rm{d}}}^{-1})=(\mathrm{Ln}\,{{\rm{N}}}_{2}-\,\mathrm{Ln}\,{{\rm{N}}}_{1})/{{\rm{t}}}_{2}-{{\rm{t}}}_{1}\\ {\rm{where}},\\ {{\rm{N}}}_{2}={\rm{Chl}}-{\rm{a}}\,({\rm{mg}}/{\rm{L}})\,{\rm{at}}\,{{\rm{t}}}_{2}\,{\rm{time}}\\ \,{{\rm{N}}}_{1}={\rm{Chl}}-{\rm{a}}\,({\rm{mg}}/{\rm{L}})\,{\rm{at}}\,{{\rm{t}}}_{1}\,{\rm{time}}\\ {{\rm{t}}}_{2}\,{\rm{and}}\,{{\rm{t}}}_{1}={\rm{times}}\,{\rm{within}}\,{\rm{the}}\,{\rm{exponential}}\,{\rm{phase}}\end{array}$$

### Pulse amplitude modulation fluorometer measurement of immobilized algal and suspension algal on ITO-based BPV devices

Photosynthetic parameters were determined fluorometrically using a Diving-PAM (Walz, Germany)^32^. RLC was obtained under software control (Wincontrol, Walz). Red light emitting diodes (LEDs) provided the actinic light used in the RLC at the level of 0, 161, 272, 400, 546, 800, 1077, 1595 and 2282 µmol photons m^−2^s^−1^. The immobilized algal and suspension algal culture in devices were first dark-adapted for 15 min before the measurements. To check if the algae were stressed by the exposure to light, maximum quantum efficiency (F_v_/F_m_) which can show the physiological state of phytoplankton was employed: F_v_/f_m_ = (F_m_ − F_0_)/F_m_ where F_m_ is the maximum fluorescence and F_0_ is the minimum fluorescence resulting in the variable fluorescence F_v_. From the initial slope (α) of the RLC, the maximum photosynthetic efficiency was obtained. The relative electron transport rate (rETR) was evaluated by multiplying the irradiance by quantum yield measured at the end of each light interval. The RLC consists of eight consecutive actinic light with increasing intensity and ten-second intervals. The photoadaptive index (E_k_) was determined from the curve fitting model^46^. The interception point of the α value with the maximum photosynthetic rate (rETR_max_) is defined as: E_k_ = rETR_max_/α. All these statistical analyses were performed using the Statistica 8 program.

### BPV Set-up and electrical measurement using immobilized algal and suspension algal in ITO coated glass devices

**Figure 4:**
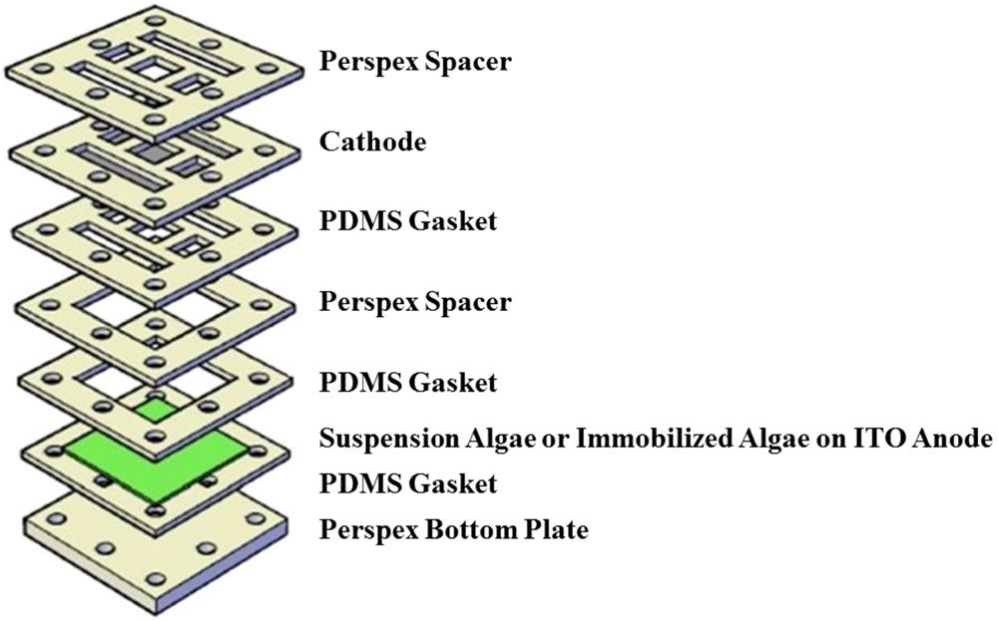
Exploded view of a BPV device with suspension algae or immobilized algae grown on ITO anode.

The closed, single-chamber BPV consisted of a (50 × 50) mm platinum-coated glass cathode was placed in parallel with ITO coated glass. Immobilized algal was grown on the surface in a clear Perspex chamber sealed with polydimethylsiloxane (PDMS) and then filled with fresh medium (Bold’s Basal medium) (Fig. 4). The body of the open-air, single-chamber BPV was constructed of clear Perspex^15^. Crocodile clips and copper wire were used to connect the anode and cathode to the external circuit. The BPV was maintained at 25 °C with irradiance of 30 μmol photons m^−2^s^−1^ for the duration of the experiments. Current outputs were measured using a multimeter (Agilent U1251B). Polarization curves were constructed by applying external resistance stepping technique for each strain which alters the resistance (10 MΩ, 5.6 MΩ, 2 MΩ, 560 KΩ, 240 KΩ, 62 KΩ, 22 KΩ, 9.1 KΩ, 2.7 KΩ and 1.1 KΩ) loads to the external circuit and by using Ohm’s law, the curves can be derived. The maximum current density and power density can be then evaluated from the polarization curve^47^. All experiments were conducted in triplicates. All statistical analyses were performed using the Statistica 8 program.
